# I am a scientist: Overcoming biased assumptions around diversity in science through explicit representation of scientists in lectures

**DOI:** 10.1371/journal.pone.0271010

**Published:** 2023-07-07

**Authors:** Dominic Charles Henri, Kirra Coates, Katharine Hubbard

**Affiliations:** Department of Biological and Marine Sciences, University of Hull, Hull, United Kingdom; Iuliu Hațieganu University of Medicine and Pharmacy: Universitatea de Medicina si Farmacie Iuliu Hatieganu, ROMANIA

## Abstract

The lack of diversity in Science, Technology, Engineering, and Mathematics (STEM) is a significant issue for the sector. Many organisations and educators have identified lack of representation of historically marginalised groups within teaching materials as a potential barrier to students feeling that a Science, Technology, Engineering, and Mathematics (STEM) career is something that they can aspire to. A key barrier to addressing the issue is providing accessible and effective evidence-based approaches for educators to implement. In this study, we explore the potential for adapting presentation slides within lectures to ‘humanise’ the scientists involved, presenting their full names and photographs alongside a Harvard style reference. The intervention stems from an initial assumption that many formal scientific referencing systems are demographic-neutral and exacerbate prevailing perceptions that STEM is not diverse. We adopt a questionnaire based methodology surveying 161 bioscience undergraduates and postgraduates at a UK civic university. We first establish that students project assumptions about the gender, location, and ethnicity of the author of a hypothetical reference, with over 50% of students assuming they are male and Western. We then explore what students think of the humanised slide design, concluding that many students see it as good pedagogical practice with some students positively changing their perceptions about diversity in science. We were unable to compare responses by participant ethnic group, but find preliminary evidence that female and non-binary students are more likely to see this as good pedagogical practice, perhaps reflecting white male fragility in being exposed to initiatives designed to highlight diversity. We conclude that humanised powerpoint slides are a potentially effective tool to highlight diversity of scientists within existing research-led teaching, but highlight that this is only a small intervention that needs to sit alongside more substantive work to address the lack of diversity in STEM.

## Introduction

It has long been recognised that there is a lack of diversity within Science, Technology, Engineering, and Mathematics (STEM) compared to the general population, and that this lack of diversity represents a loss of talent within the sector. A report in 2020 into the UK STEM workforce identified that 65% of STEM professionals are white men and that women are particularly underrepresented [1]. Other measures of success in science show similar bias. For example, 90% of Fellows of the UK Royal Society are male [2]. Researchers from Black, Asian, and Ethnic Minority backgrounds are less likely to be awarded governmental research funding and are funded with smaller grants [3]. Lesbian, Gay, Bisexual, and Queer students are less likely to complete a STEM qualification than their heterosexual peers [4]. There is evidence to support the idea that diversity is beneficial to scientific productivity [5]. Nationally and ethnically diverse research teams have been found to produce more papers with higher impact [6–8]. Gender-diversity with research teams has led to important changes of perspective within multiple fields and an increased focus on under-researched topics [9, 10]. However, several studies have shown that implicit stereotypes about who belongs in certain STEM careers negatively influence the hiring, salaries, and promotion of women relative to men in STEM careers [11–13]. Loss of talented individuals from the STEM workforce is a matter of significant concern, and proactive measures are increasingly being put in place to attract and retain diverse members of the scientific community.

### A white male Western bias persists throughout formal STEM education

In order to become a STEM professional an individual must view a technical career as something that they want and can realistically achieve. They must also be able to see themselves working in STEM and adopting a scientific identity [14, 15]. Assumptions about who can be a scientist are established at a young age and persist throughout education. The Draw A Scientist Test (DAST) is a commonly used methodology to explore children’s conceptions of scientists used internationally since at least 1957 [16–18]. Children typically draw scientists as male, bald/bearded, wearing a lab coat, and performing chemistry related tasks [16, 18]; although studies between 1985 and 2015 have found that this masculine bias has declined [18]. Children are more likely to draw male scientists as they grow older [18], suggesting that masculine stereotypes of scientists are reinforced through formal school education. Teaching resources may reinforce this perception that scientists are white and male. For example, a recent analysis of high-school level chemistry textbooks used in three different countries found a significant male bias in the scientists presented [19]. Representations of men tended to be as active scientists, whereas women were more likely to be presented in non-scientific contexts such as domestic settings [19]. Some evidence suggests that there has been little progress in improving representation within textbooks since earlier analyses in the 1970s and 1990s [20, 21]. Other evidence suggests that female representation within textbooks is increasing in line with the proportion of females within the field [22]. However, studies generally agree that the ethnicity, gender, disability status, nationality, sexual orientation, and socioeconomic representation of scientists in taught materials does not match that of the student body [22–24]. These biases persist into undergraduate and postgraduate education. For example, in the UK there are relatively fewer Black, Asian, and Ethnic Minority postgraduate students in science than at undergraduate level [25]. This means that the pool of graduate teaching assistants will look less diverse than the undergraduate class. Recommended reading lists in science have attracted less attention than in the arts and humanities, but there is some evidence to suggest that science undergraduates are disproportionately directed towards literature from male authors and to few studies conducted outside of Europe, Australia and North America [24]. Exposure to these repeated biases throughout education reinforce a norm that scientists are more likely to be white, male, able bodied, and Western.

The lack of diversity in the way that science is traditionally presented means that learners may not have visible role models available to them. As such, learners from historically minoritized groups may feel excluded from science or that scientific careers are not something that they can aspire to [26]. Much of the thinking around scientific role models has centred on binary gender representation but representation of ethnicity, disability, and LGBTQ+ identity is gaining increasing focus [26–29]. Having visible role models from a similar background may increase a student’s sense of science identity [30]; i.e. a student’s sense of themselves as the kind of person who can succeed in STEM [31]. For example, faculty members can act as positive role models for their students. There is evidence to suggest that being taught a traditionally male-biased STEM subject by female instructors increases the likelihood that a female student chooses to continue studying that subject [32, 33]. Similarly, having an instructor from a similar ethnic background increases the educational performance of Black students [34]. However, if the teaching staff in a given department are not particularly diverse, relying on faculty members as role models will be insufficient and more proactive efforts need to be made to increase representation.

### Barriers to improving representation in university level STEM education

While the lack of diversity in science is increasingly seen as an issue that needs to be confronted, individual educators often struggle to identify tangible actions they can take to address this within their teaching [35]. There have been many calls to diversify and decolonise science teaching [36], actively confronting the historical legacies of science and the white Western approaches that underpin scientific thinking and conventions [27]. Decolonisation and diversification is an issue for all disciplines but is often seen as something that is more relevant for arts and humanities than the sciences. Many faculty members actively disagree with diversification efforts within science, seeing science as universal and inherently objective [37]. This mindset fails to acknowledge that science is built on white Western forms of knowledge and thought, and that this bias might be alienating to students of colour [27]. The objectivity argument also fails to account for implicit biases about the quality of science from Lower and Middle Income countries [38]. Even well-meaning teaching staff in science subjects often feel that they cannot devote time to diversity and inclusivity within the curriculum due to the amount of technical content to be delivered [39]. Given the historical white male bias of scientific research, the need to cover key topics in the development of the discipline may hinder efforts to present a greater diversity of scientists. Scientists also often lack the confidence and training to engage with this due to their lack of background in historical, cultural, or social science disciplines [40]. Many academics also question whether they are even allowed to engage in these discussions when they do not belong to historically marginalised groups or assume that responsibility for inclusion lies elsewhere in the university [39]. The burden of addressing equity and diversity issues often falls disproportionately to faculty members from historically marginalised groups, creating an unfair burden on individuals who are already structurally disadvantaged by the academy [41–43]. To avoid this, all members of the academic community have a responsibility to actively address equity and diversity. Individual academics, including those from majority demographic groups, need to feel empowered to take tangible actions and to recognise that they have an important personal role to play in establishing an inclusive learning environment [35, 39].

### Strategies faculty members can used to increase awareness of equity, diversity and inclusion into disciplinary teaching

There are multiple strategies that could be used to embed consideration of equity, diversity, and inclusion into university curricula. For example, some instructors are now introducing implicit bias tests such as the Harvard Implicit Association Test (IAT) [44] into the curriculum and asking students to reflect on their biases and assumptions [45–48]. This strategy has been adopted in several healthcare disciplines but there are recent reports of similar strategies being used within STEM. One US based chemistry academic describes a positive effect of introducing extra credit activities such as taking the Harvard IAT and writing a reflective essay based on the bias highlighted [49]. However, incorporation of these activities takes up time in the curriculum and may be perceived by students as irrelevant to their scientific training. Some students may go further and respond to diversity-related interventions with hostility; a concept found inside and outside of Higher Education often termed white and/or male ‘fragility’ [50, 51]. It is worth advertising at this stage that good resources with actionable interventions to improve the inclusivity of university teaching are available (see Dewsbury and Brame [52] and Hubbard and Gawthorpe [53]).

An alternative approach that is more naturally compatible with delivery of science teaching is to introduce more explicit representations of diversity within existing lectures. One advantage of a research-driven curriculum within universities is that teaching materials like presentation slides produced by individual members of teaching staff can change much more dynamically than resources such as textbooks. Case studies of individual scientists from a variety of backgrounds could be included [30], or a diverse range of guest experts invited to contribute to taught sessions [54, 55]. These strategies are potentially powerful but again require space to be found in the curriculum or rely on academics personally knowing individuals from a range of backgrounds and identities they could invite, so may be difficult to implement. Alternatively, faculty could actively incorporate research from a more diverse authorship into their teaching. There are some efforts to provide resources banks to help academics with this in a subject specific context; for example, Project Biodiversify is an emerging collection of case studies and resources of scientists from a variety of different backgrounds [56]. While this is to be encouraged, academics may still be concerned that incorporation of more diverse authors may result in even more content being added to crowded curricula or result in less coherent summaries of disciplines if key references are removed to accommodate diversity of authorship [37].

**Fig 1:**
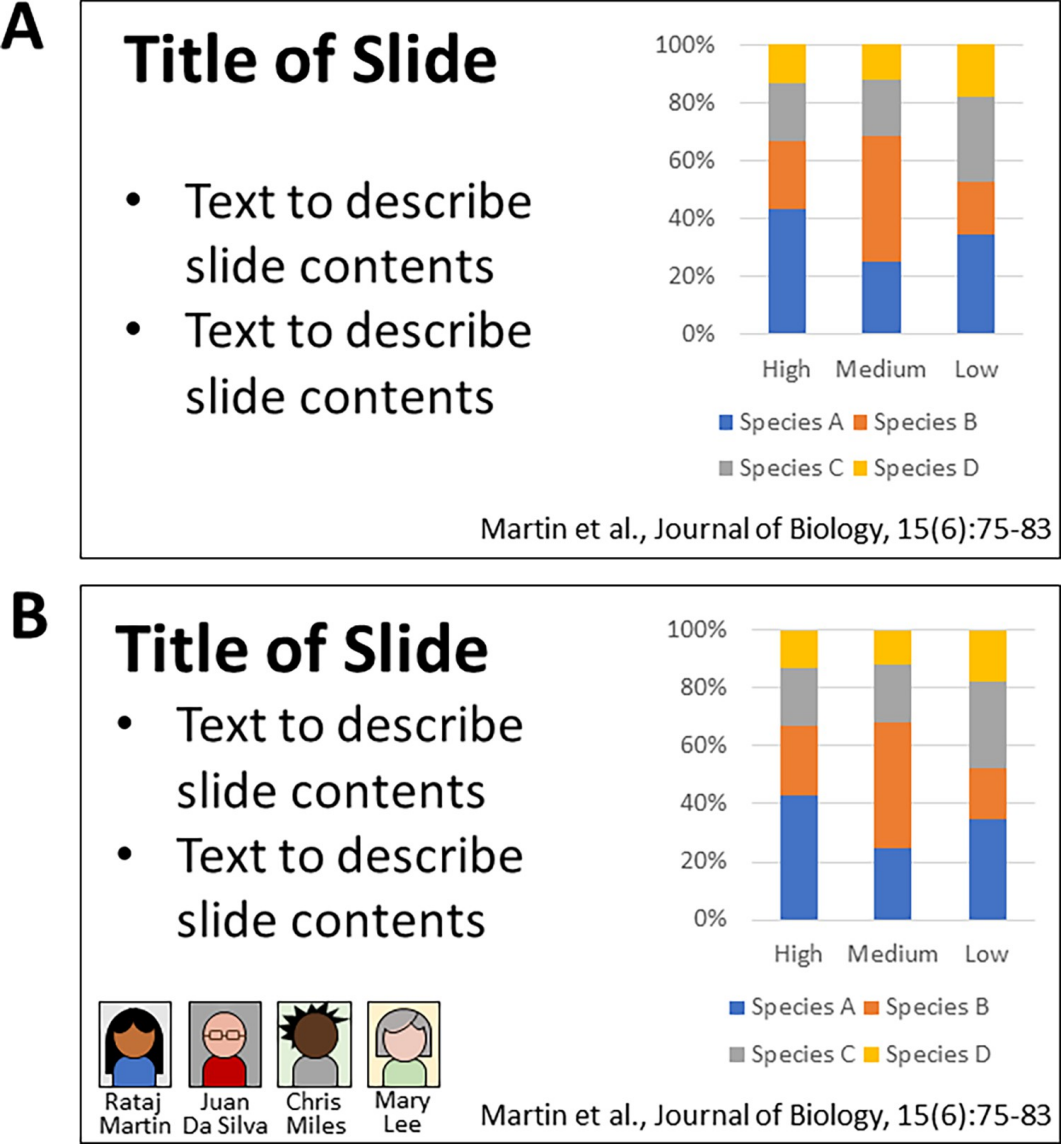
Stylized representations of traditional and humanised powerpoint slide designs. A: Traditionally formatted scientific slide, with author information presented only as a formal citation. B: Humanised slide, with photographs and full names of authors provided to give more explicit representation of author identity. Stylized slide presented for a fictional citation, dataset, and author list; note that real lecture slides included photographs rather than the cartoon style representations shown here.

A simpler alternative is to better represent the diversity of authors of papers that are already included within existing lecture materials. For example, a typical powerpoint slide might present a scientific result (e.g. a graph) alongside a formal citation of the work using a standard referencing convention such as Harvard style (Fig 1A). While this format is frequently used, it potentially dehumanises the scientists involved and obscures any demographic information such as gender (identity), ethnicity, age, or other observable characteristics [35]. As such, we hypothesise that students may under-appreciate the diversity of practising scientists when research is presented in this format, instead relying on learned biases about the authors potential identity. This study therefore considers the impact of including photographs and full names of authors on existing powerpoint slides, giving a humanised representation of the scientists. In this format, students are presented with explicit representation of scientists alongside their findings and citation (Fig 1B). This is easy to implement, requiring only relatively minor modifications to existing teaching materials. It does not require time to be spent actively discussing diversity in class, but increases the diversity of representation that students are exposed to while learning about current research. While this method has already been adopted elsewhere [56], to the best of our knowledge no previous studies have attempted to investigate the impact of the intervention or students’ perceptions of it.

### Aim of current study

This study aims to explore the impact of presenting photographs and full names of scientists on existing lecture slides as an educational practice that could be easily adopted more widely. We present the results of a survey completed by 161 bioscience undergraduate and taught masters students at a civic university in the United Kingdom. The study has five specific research questions:

1. Do students make assumptions about the identity of scientists based on presentation of author information through formal referencing conventions?
2. Do these assumptions align with the biased representation of diversity in STEM found in the wider literature?
3. Does presentation of photographs and full names of authors of scientific papers change student perceptions of diversity in science?
4. Do students think that presentation of photographs and full names alongside formal referencing conventions is good educational practice?
5. Does the gender or ethnic group of the participant have an influence on any of the above?

### Positionality statement and study context

This study was conducted at a medium sized civic university in the north of England. Students at this university are predominantly female (58%), white (86%), and 18% have declared a disability [57]. The study was conducted with students across several modules within the Biological and Environmental science subject groups (Table 1). The gender and ethnicity ratios of our survey population closely match that of the wider university; however, we acknowledge that this gives our sample a considerable white bias compared with other studies conducted in more diverse institutions. Within the Biological Science and Environmental Sciences staff at this institution, the gender ratio is 62% male: 38% female and the vast majority of teaching staff are white. All authors identify as white British. Two of the authors (DH and KH) were instructors on modules from which study participants were drawn, and all surveys were conducted during sessions taught by DH and KH. Some of the modules were core modules for all students, while others were optional. Our participant pool was drawn entirely within the context of biosciences students, which means that our study may not be broadly applicable to STEM disciplines because of documented differences in representation of diversity across the STEM spectrum [58]. Both DH and KH are actively involved in university initiatives to improve diversity and representation within STEM, so the use of humanised slides described in this study occurred within a broader context of diversification of the disciplinary curriculum.

**Table 1:** Survey population and response rates.

Level of study	Year of Course	Module Title	Academic Year	Number of students on module	Number of survey participants	Response Rate (%)
Undergraduate	1st	Cells and Organelles	2020/21	150	58	39
	3rd	Insect Biology	2019/20	53	28	53
	3rd	Applied Ecology	2019/20	38	9	24
	3rd	Wildlife Ecology and Management	2020/21	33	6	18
	3rd	Wildlife Ecology and Management	2021/22	29	10	34
Postgraduate Masters		Environmental Change in the Anthropocene (ECiA)	2019/20	18	5	28
		ECiA	2020/21	26	16	62
		ECiA	2021/22	50	29	58
Total Undergraduate				303	111	37
Total Postgraduate				94	50	53
**Total all students**				**397**	**161**	**41**

## Methods

### Ethical oversight

Ethical oversight for this study is provided by the University of Hull Faculty of Science and Engineering Ethics committee (Project code FEC_2019_204). Participation in the study was entirely voluntary, and participants were provided with study information before providing informed consent.

#### Questionnaire distribution

This study was undertaken both before and during the COVID19 pandemic (March 2020 to November 2021). This is relevant because the primary mode of delivery of lecture-based teaching changed during this period of time from face-to-face and didactic to online and flipped. However, the fundamental concept of the survey remained the same as it consisted of a total of eight questions and used a pre / post format centred around a single taught session on a course (with the method repeated across multiple individual sessions on multiple courses). Participation was entirely voluntary with no incentivisation and students were asked to not submit a survey if they had done so previously elsewhere on the course. Prior to the taught session, participants completed the four questions investigating their scientific self-identity, their sense of belonging within the scientific community, and the implicit bias test. After the taught session, participants completed the four questions related to the humanised slide intervention. Inclusion of demographic information on gender and ethnic group was an optional extra at the end of the survey. Before the pandemic surveys were paper-based, with the pre and pos’ questions on different sides of the same sheet. During the pandemic surveys were presented as two separate Canvas (Virtual Learning Environment) quizzes separating out the pre and post questions.

#### Survey questions

Prior to the beginning of a taught session, participants were asked to share their first impressions on the identity of the author of a hypothetical reference in Harvard format; Lee, M. (2019) ‘Globally interesting Biology’. *Biology Journal*: 45 p. 12–25. ‘M. Lee’ was chosen specifically to allow for multiple and diverse assumptions about the author as the surname Lee is common in communities descended from Anglophone, Korean, and Chinese ethnicities. There are many first names beginning with ‘M’ through most ethnicities with limited inherent bias as to the gender of the name (See S1 File for further justification of the choice of name). Participants were asked specifically to reflect on “what the author looks like, their first name, and where they come from?”. To the best of our knowledge, no other study has undertaken an investigation similar to this and, therefore, there may be uncertainties about using an unvalidated tool. However, as much as possible the question was designed to not lead the participants to any particular response. In fact, in the results section many responses did make explicit assumptions about the author but a large proportion of participants highlighted that one cannot make assumptions based on the information provided. The survey followed a straightforward approach to gaining student perspectives on the humanised slide intervention. Participants were asked whether the intervention had changed their perspective on their answers to the set of questions in the pre survey. They were then asked whether they felt that having humanised slides could be considered good practice. All survey questions can be found in full in Table A in S1 File.

#### Thematic coding

To analyse the free text data qualitatively, we undertook a thematic analysis based on the protocols outlined by Braun and Clarke [59]. Thematic analysis is a widely used, flexible, and rigorous approach to analysing qualitative data through the development of themes and subthemes within a dataset [59, 60]. Two members of the research team coded the data; DH did the initial coding which was agreed by KH.

As we were coding to a relatively straightforward deductive framework (e.g. positive, negative or ambivalent response, or assumptions based on previous DAST studies outlined above) there was relatively little ambiguity of how responses should be coded. In the instances that there were ambiguous responses, they were discussed between the research team to reach a consensus. This coding structure was developed to ensure that every single response could only be placed into one of the potential sub-themes within each theme; i.e. that they were mutually exclusive. For example, the response “White male, middle aged, Mark??? England or America’’ includes clear statements that identify ‘Bias’ for all author characteristics and coding to any of the other sub-theme for each author characteristic would be incorrect. If an assumption about a specific characteristic was not included in the response, it would be coded as ‘Not Considered’. This means that for the responses analysed by chi-squared analysis, no response could be coded into more than one subtheme; e.g. No response could be both ‘Bias’ and ‘Diversity aware’ regarding the author’s ethnic identity. Decisions regarding what was allocated to the bias category were based on the stereotypical representation of scientists in DAST studies (i.e. Male, White, and Old [bald/bearded] [16]), with the addition of Western to represent the widely reported Western-centric focus of Higher Education curricula. We specifically made the decision on the term reverse-bias to represent any assumptions that did not align with the points above because reverse-bias does not necessarily denote a positive response, any form of bias can still be tied to harmful stereotypes around who belongs in science [61]. The exact terms that define each theme are outlined in Supplement A in S1 File.

The mutual exclusivity of the coding themes means that the data meet the assumptions for a chi-square test of independence, with the theme of the response being a categorical dependent variable, and gender/ethnic group being categorical independent variables [62]. For each question, two chi-squared tests were performed to determine whether the proportions of responses coded to each of the themes were altered by participant gender or ethnic Group (one test for each categorical variable). Bonferroni corrections were used to account for the use of multiple tests per question. Note that not all participants provided demographic information, and responses that left this blank or responded prefer not to say were not included in these analyses. Due to limited representation in the data set, gender and ethnic Group variables were aggregated into two categories centred around an analysis of the concept of white, male fragility [51]. Participant gender was represented as either Male or Female and Non-binary. Ethnic group was separated into White or Black, Asian, or Mixed Ethnicity (i.e. BAME). However, even with this correction there was insufficient representation within the BAME group for statistical tests investigating ethnic group as a predictor variable.

## Results

Biosciences students on eight different taught modules where the humanised slides were used were invited to complete the survey. A total of 161 Biology, Zoology, and Ecology students responded to the survey, including 111 undergraduates and 50 students on taught masters courses (Table 1). This represents a 41% response rate, so findings are not necessarily reflective of the whole cohort. Within the 161 respondents, 125 provided their gender identity, and 124 provided their self-identified ethnic group (Table 2). Of those that provided demographic information, there was a bias towards female (58%) and white (87%) participants. As there were relatively few responses from Black, Asian and Minority ethnic group students these have been grouped together for analysis, however we recognise that this may obscure differences between cultural groups. We also grouped together female and non-binary students for analysis, but excluded those who preferred not to disclose their gender identity from the statistical analyses.

**Table 2:** Participant demographics.

Demographic	Category	Number of responses	% of total responses	% of those that provided demographic information
Gender	Female	73	45	58
	Male	48	30	38
	Non-binary	2	1	2
	Prefer not to say	2	1	2
	Not provided	36	22	-
Ethnic Group	White	108	67	87
	Black, Asian or Mixed Ethnicity	16	10	13
	Not provided	37	23	-
Total		161	*-*	*-*

### Student assumptions of author identity

We first wanted to determine if students held assumptions around author identity when presented with a conventional academic reference. There were 136 understandable and complete responses to the survey question asking students to share their first impressions on the identity of the author of a hypothetical reference in Harvard format; Lee, M. (2019) ‘Globally interesting Biology’. *Biology Journal*: 45 p. 12–25. All responses were coded to one of four categories for each of four different characteristics according to whether they aligned with widely reported biases in the representation of scientists. The categories were bias, reverse bias, not considered, and diversity assumed. The characteristics were gender (bias = male/man), geographic location (bias = Western-centric), ethnicity (bias = white), and age (bias = old/middle-aged).

“*White male*, *middle aged*, *Mark*??? *England or America*”—an example of a response coded as ‘Bias’ for all four characteristics.

**Fig 2:**
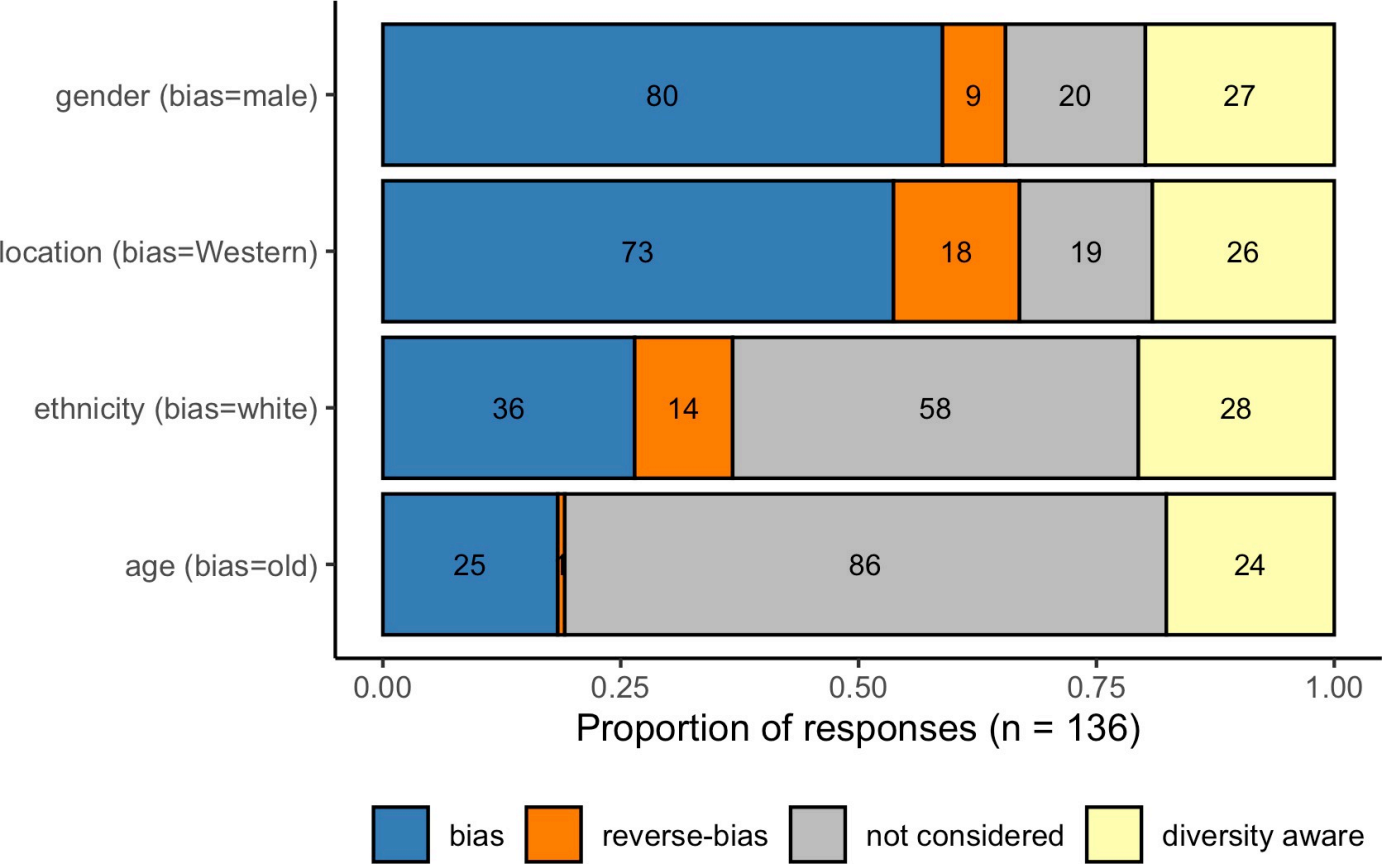
Assumptions of author identity around gender, location, ethnicity, and age made from a Harvard style reference. The bias responses (e.g. male, white, Western, old) are presented in blue, reverse-bias responses (e.g. female, Black/Asian, non-Western, young) in orange. Diversity aware responses are those where students either presented different interpretations considering multiple diverse identities or actively said that one could not infer this information from the citation.

The most common assumptions students made about author identity related to gender and location (Fig 2). Of the 136 responses, 59% explicitly assumed that the author was male, and 54% assumed they were from a Western-centric country (i.e. USA, Europe or Australia). Participants were less likely to make explicit statements about the ethnicity of the author, but the most common explicit assumption was that the author was white (26% of all respondents). White ethnicity was only coded when the terms white or caucasian were used and not based on location assumptions, so the difference in location and ethnicity data may be due to a lack of clarity in the format of communication. Author age was not asked for, but many participants made references to the age of the author, as well as regular references to facial hair. Age was the least frequently mentioned aspect of author identity, but the most common assumption was that the author was old or middle-aged (18%).

Reverse-bias responses were most common for location 13% (i.e. not Western), then ethnicity 10% (i.e. not White), then gender 7% (i.e. not male), and one respondent explicitly stated the author was young (i.e. not middle-aged or old). It is worth noting that all but one of the responses coded to reverse bias for location and ethnicity assumed that the author was “Asian”; mostly commonly Chinese or Chinese / Korean. One respondent stated that they thought the author’s first name was Mohammed and assumed they were Arabic. Respondents who did not make explicit assumptions either said that they were unsure about author identity, or that one could not make assumptions about an author from a Harvard reference; which we coded as ‘Diversity aware’. Diversity aware responses were given by approximately 20% of participants (age 18%, location 19%, gender 20%, and ethnicity 21%). However, within diversity aware responses some participants still made assumptions about the identity of the author. For example, one response stated that one cannot make assumptions about ethnicity or location, but then uses masculine pronouns demonstrating implicit gender bias; “*There isn’t a first instinct*, *name can’t be used to distinguish what someone looks like or where they come from*. ***He’s*** *just as likely to be a white american as* ***he*** *is to be a black african*”.

Within the responses that did make assumptions about author identity, the majority of responses represented the bias response. The strongest bias was for age (96% biased: 4% reverse-biased), followed by gender (90%:10%), location (80%:20%), and ethnicity (72%: 28%).

**Fig 3:**
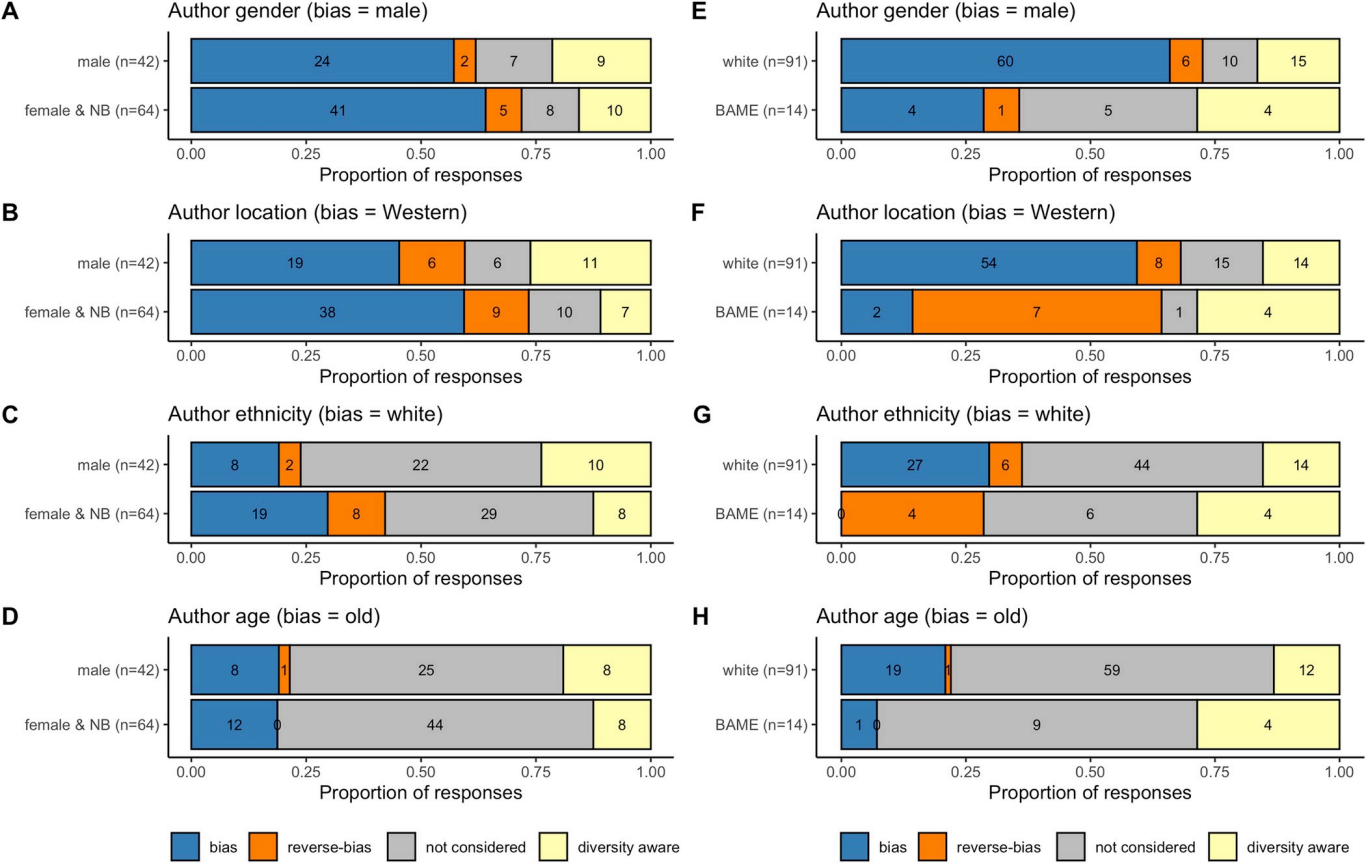
Breakdown of responses to Harvard style reference by participant gender (A-D) and participant ethnicity (E-H). NB = Non-binary, BAME = Black, Asian, and Minority Ethnic. The implicit bias responses (e.g. male, white, Western-centric, old) are presented in blue, reverse-bias responses (e.g. female, Black/Asian, non-Western, young) in orange. Diversity aware responses are those where students actively said that one could not infer this information from the citation.

We were interested to see if the personal characteristics of the students made any difference in their assumptions about author identity. We broke down the responses to the assumptions data by participant gender and ethnic group (Fig 3). There was insufficient data to reliably perform statistical analysis of responses by participant gender or ethnic group, as for all comparisons there were multiple categories with fewer than 5 respondents making a Chi-square test inappropriate. However, it should be noted that none of our BAME participants assumed that the author was white (Fig 3G), and the majority of BAME participants gave the reverse-biased response for location (in all of these instances ethnicity assumptions were Asian or Chinese; Fig 3F). Our data indicates that a majority of students from all backgrounds do make assumptions about who is participating in science from Harvard style references. However, the extent to which these assumptions align with widely reported biases in the representation of diversity in STEM might differ between students from different demographic groups.

### Impact on participants perception of diversity in biosciences

**Fig 4:**
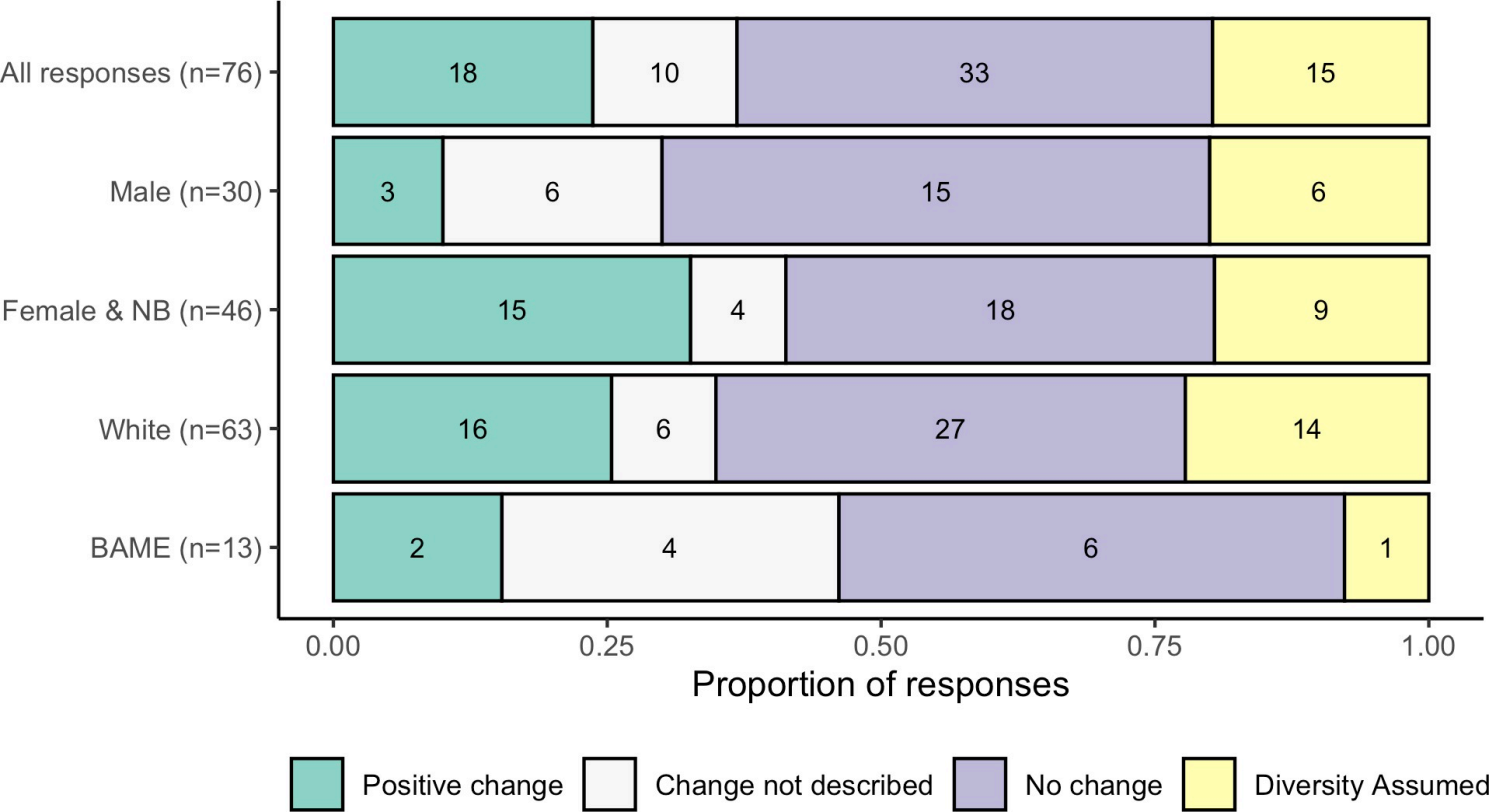
Participant perceptions on whether the ‘humanised’ slide design impacted their perception of diversity in science, broken down by gender and ethnic group of the participant. NB = Non-binary, BAME = Black, Asian, and Minority Ethnic.

Having established that students did make assumptions about author identity from Harvard style references and that these assumptions align with widely reported biases, we then investigated the impact of using the humanised slide design in the lecture. We asked students whether seeing explicit representations of scientists changed their perceptions of diversity within science (Fig 4). There were 76 coherent responses which were all coded as either ‘Explicit no change’, ‘Explicit positive change’, ‘Diversity assumed’, and ‘Change not described’. The most commonly coded response was ‘Explicit no change’ (33/76), mostly without further justification (i.e “*No*”); although some responses outlined that the participant felt the practice was irrelevant and/or unnecessary (3/33). Of the 33, ‘Explicit no change’ responses, five suggested that the practice was insufficient or did not display enough diversity. Interestingly within this category, two participants’ responses were very focused on gender as diversity and less receptive to ethnicity/location as diversity (e.g. “*No*, *majority were still male (although more diverse) -> mainly asain [sic]*”). Note that the claim that the “majority were male” is inaccurate as a minimum all lectures were designed to present at least 50% of the scientists as female.

The second most commonly coded response was ‘Explicit positive change’ (18/76) where participants stated that their perceptions of diversity in the field was improved; most often this was gender-related but students also commented on how their perceptions of ethnicity, gender-identity, and/or age were impacted (e.g.“*Yes—it highlights the presence of ’non-cis white males’ also publishing papers*”). A common sub-theme within the ‘Explicit positive change’ theme, was participants expressing the belief that there is no standard look for a scientist or that anyone can be a scientist (6/18) (e.g. “*There isn’t a certain "look" for a biologist—anyone can be one*”).

The final theme that was expressed consistently throughout the responses was the idea that the practice did not change the participant’s perceptions because they already knew that the field was full of diversity (diversity assumed) [15/76] (e.g. “*Not at all*, *anyone can be a biologist like anyone can cook*”). Five respondents in the diversity assumed category made explicit/implicit assumptions about the identity of the hypothetical author earlier in the survey and then said that one could not make assumptions (e.g. “*First name*: *Michael*. *From*: *Europe*” followed by “*No*, *I already had the mindset that it would be a wide range of people carrying out the research*”). The remaining responses were either attempts at humour, expressing a lack of understanding of the question, or expressing disinterest in the survey, which were all coded as Change not described. There was no difference in the proportion of responses coded in each of the four categories according to participant gender (Fig 4; X^2^_1(N = 77)_ = 6.17, p = 0.10). The low number of responses from BAME students meant that statistical comparison is inappropriate, but the data is presented in Fig 4.

### Student perceptions of the practice

We investigated whether students felt that there was value in explicit representations of diversity in lectures as an educational practice by asking whether they felt it was good practice. There were 91 coherent responses which were all coded as either ‘Explicit good practice’, ‘Ambivalence’, or ‘Explicit not good practice’.

The most commonly coded response expressed explicit support for the practice (68/91 = 74.7%). While 18 of these responses were positive with no additional details (e.g. *“Yes”*), the vast majority of respondents made some attempt to explain their decision. This elaboration was further broken down into a number of sub-themes evident in Table 3; note that responses could be coded in multiple sub-themes but not in multiple themes. ‘Explicit good practice’ responses were most commonly qualified with statements expressing the value of the practice for raising awareness of diversity in science/bioscience. There was also a related but subtly different theme expressing that the practice humanised the researchers and/or made them more personally relatable to the participants. Some humanised responses focused on how the practice might help other people, while for others it had a more personal impact on how they felt they fit within STEM.

**Table 3:** Frequencies of sub themes within the explicit support for practice, presented by participant gender.

Sub theme	Illustrative examples	Male (n = 25)	Female + Non-Binary (n = 41)	Total (n = 68)
‘Awareness of diversity’	“*Yes*, *breaks down stigma that you have to fit a specific mold to be successful in science*” *“Absolutely—yes*. *Using pictures and full names ’normalises’ the huge variety of people who contribute science to society*. *This provides a platform for equality and inclusivity and makes people think they can do science too—which of course they can”*	7 (33%)	27 (60%)	34 (50%)
‘Humanising researchers’	“*Seeing a face and a name behind the research*, *makes it less intimidating*, *and potentially more reader friendly*” “*Seeing just one visibly queer person would help me i think*. *nothing against old white men but it would help me feel safe*”	5 (24%)	1 (31%)	19 (28%)
‘Credit + recognition’	“*Yes*, *they work hard and deserve to be recognised*” *“Yes*, *it give’s more perspective over when their research was carried out and makes me feel more invested in their research as oppose to one of thousands of relatively anonymous scientists as they aren’t appraised enough for their contribution”*	7 (33%)	2 (4%)	9 (13%)
‘Utilitarian’	“*Yes*, *gives us more knowledge for future work we may have to look into that could be linked”* *“Yes*, *can tell exactly who wrote the article and easier to find it for own reading”*	5 (24%)	7 (16%)	12 (18%)

There were some additional and unexpected reasons why respondents felt the practice was positive that were unrelated to diversity in STEM (Table 3). The idea of credit, praise, or recognition for the scientists undertaking the work was a recurring sub-theme, focused on the idea that scientists deserved less anonymity in their contribution to the field. The final sub-theme expressed that having full names and headshots might help them remember the content of the papers better or find more research by the same authors, which was coded as ‘Utilitarian’. Initial investigation of these sub-themes by participant gender suggests that male participants were more likely to focus on credit and recognition than female and non-binary students (33% of male responses, 4% of female/non-binary). Conversely, female and non-binary participants were more likely to provide responses aligning to the sub-themes ‘awareness of diversity’ (60% of female/non-binary, 33% of male) and ‘humanising researchers’ (31% of female/non-binary, 24% of male). Note that while a gender bias is suggested in the qualitative data, because each response could be categorised in multiple sub-themes statistical analyses are inappropriate.

**Fig 5:**
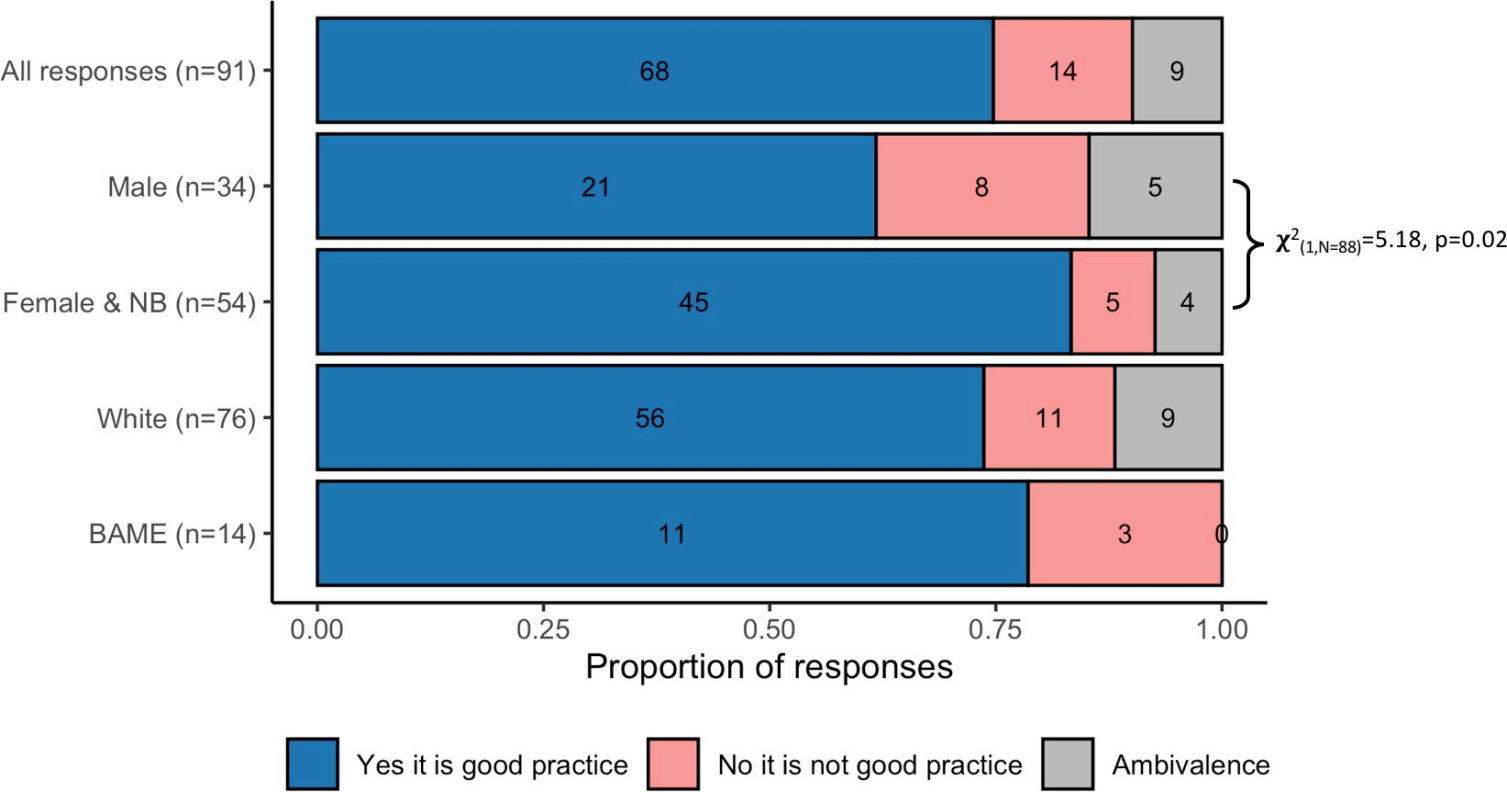
Student perceptions on whether it is good practice to show headshots and full names of all scientists in lectures, according to the gender of the participant. Annotation indicates the result of a Chi-square test to compare perceptions by gender. To avoid cell values smaller than five the ‘Not good practice’ and ‘Ambivalence’ categories were aggregated for the statistical analysis. Comparatively low representation of BAME participants mean the equivalent test for participant ethnic group was inappropriate but data are presented for interest.

A much smaller number of respondents disagreed that this was good practice (14/91), coded as Explicit not good practice. Justifications sometimes included that the practice was unnecessary or detracted from the science (e.g. “*I would rather focus on the information rather than the author*”). A further nine respondents were unsure, ambivalent, or had mixed feelings about the practice (e.g. “*Interesting but unsure of relevance*”). A deeper investigation of student responses to this question according to the self-identified gender of the respondent suggests that males provided a significantly greater proportion of negative and ambivalent feelings about the practice than female and non-binary students (Fig 5; X^2^_1(N = 88)_ = 5.18, p = 0.02). The low number of responses from BAME students meant that statistical comparison is inappropriate, but the data is presented in Fig 5.

## Discussion

We found that most participants made some kind of assumption about the gender, ethnic group, location, or age of a hypothetical paper author cited in the Harvard style; with clear biases towards the author being male, Western, white, and middle-aged or older (Fig 2). These assumptions about who researchers are match those present in validated measures of implicit diversity biases in STEM, such as DAST [16–18] and the Harvard IAT [44]. Our results also align with the predictions of other studies looking at the diversity of STEM researchers in the taught environment like reading lists and textbooks [19]. It is worth noting that in our study white, male, and Western biases appear to be consistent across participant genders, and that even students who were consciously aware of the potential for implicit bias still made assumptions about the author’s identity. Within the dataset, there was one example of a response that was certainly inappropriate and could be perceived as racist (see Table B in S1 File), provided by a white male student. Also of concern is that, except for one respondent, ethnic group and location reverse biases were consistent with the Model Minority Stereotype purporting that Asian people have natural propensities for STEM disciplines [61]. Combined these results suggest that student implicit biases about the diversity of STEM are exacerbated by the absence of explicit diversity cues in neutral referencing systems. Stereotypes around who belongs in STEM, often perpetuated by formal education systems [23], are known to cause damage in a number of ways. Individuals within minority groups in the UK and US can feel excluded and are less likely to take up careers in STEM [1, 2, 14] or encounter slights, condescension, and insults if they do [41, 42, 63]. These same stereotypes generate unconscious biases that have been shown to hinder career progression for women in STEM [11, 13]. Even stereotypes that might be incorrectly considered beneficial, such as the Model Minority Stereotype, cause distress for people who feel pressure to conform to or rebel against the stereotype [61]. Celebrating and explicitly recognising diversity within STEM is one of the many interventions available to help address the harms perpetuated by these stereotypes [35, 42, 52].

Having established that a diversity-neutral researcher citation format facilitates biased perceptions of STEM diversity, we then asked whether our visible diversity intervention was able to challenge student perception of diversity in STEM. When asked whether the humanised slides impacted participant perceptions of STEM diversity, over 60% of the participants felt that their perceptions were unchanged (Fig 4). Some felt that the intervention was insufficient to engender change, while others responded that they had already assumed that STEM was a diverse field. However, for an important subset of those surveyed (over a fifth) the intervention was impactful, with many highlighting that it challenged stereotypes about who can be a scientist. The impact was not necessarily just felt among students within groups that are seen as under-represented within STEM, as we did not find a significant difference in impacts between male and female or non-binary students. Our sample was too small to formally compare the impact along participant ethnic group lines but this is an area that could be investigated further with a larger study.

Some of the key barriers to engagement with diversity enhancing interventions are student perceptions of them and their willingness to engage with them; particularly those students within majority groups who may feel threatened [64, 65]. However, when asked in this study, 75% of respondents to the question agreed that the use of humanised slides was good practice (Fig 5). Most commonly this was linked to the potential for the intervention to raise awareness of diversity in STEM or supporting an individual’s sense of belonging within the field, which is consistent with other studies of the use of pictures to highlight diversity in STEM [66]. In addition to those reasons expected, some students highlighted the value of the practice for providing proper recognition to hardworking researchers or for improving their ability to investigate the research more easily. There was a statistically significant gender split in the data, which suggests that male participant perceptions were more likely to be negative or ambivalent than female and non-binary participants. While the overall proportion was small and a wider study would be of value to confirm this result, this fits with the wider literature on the concepts of white, male fragility [50]. Negative responses particularly focused on the intervention as being unnecessary or irrelevant, which is well-established within the literature on barriers to anti-racist and anti-sexist pedagogies [65, 67]. This response likely stems from initiatives that highlight white and/or male privilege as being interpreted as personal attacks or as minimising the struggle and effort they have invested into their own successes [50]. Interestingly, male participants that felt it was good practice appeared more likely to focus on credit/recognition as a benefit of humanised slides while female and non-binary students were more likely to raise diversity and/or belonging values. We were not able to investigate this idea statistically and further studies are required to confirm this position. The presence of an explicit benefit to the diversity enhancing intervention that does not clash with white and/or male self-identities might help wider uptake of such initiatives. There is evidence that presenting a pedagogic practice specifically as a diversity intervention can increase resistance to its uptake [39]. In the instance of this particular study, students were able to self-construct the potential benefits of humanised slides without them being presented specifically as a diversity related intervention, which may have enhanced participant reception of the practice.

### Limitations of study

This is intended as an exploratory study to explore the impact of how we represent scientists within our lecture and whether this can challenge widely-held biases about the authors of scientific papers. While our conclusions and methodology are robust within the scope of the study, our findings are not necessarily applicable in all contexts. The sample size is modest, but compares favourably with some other published reports of interventions to improve student understanding of diversity in university STEM settings [49, 68]. While our sample is drawn from postgraduate and undergraduate students at multiple stages of their degrees, participants are all at a single UK university. Participation in this study was voluntary, resulting in a response rate around 41%, so responses may not reflect those of the wider cohort. Our data collection also happens entirely within the context of biosciences and ecology which are typically more gender balanced, so may not represent other STEM disciplines. It would be particularly informative to extend this study across the STEM disciplines, including those with a more significant gender and ethnicity bias. For example, the Geosciences have been highlighted as being particularly biased towards white researchers [69], and the significant male bias in physical sciences, engineering, and mathematics is well known [1]. Our method of investigating diversity bias by recording student projection onto a Harvard style reference is a novel research methodology which should be developed and validated further, but the white Western male bias in responses is consistent with that exposed by other well established methodologies such as the DAST and Harvard IAT [17, 18, 44]. Future studies could investigate whether the strength of bias is associated with different demographics of participants or the level of demographic skew within the discipline. It should be noted that our study captures immediate responses to the impact of the humanised powerpoint slide design, but does not attempt to measure a longer-term impact or changes in student opinions about whether they are likely to undertake a scientific career. However, we consider the research to have value as a snap shot study that highlights potential opportunities to increase diversity and representation within STEM education, as well as presenting an opportunity to have more open discussions with students about diversity within the curriculum. Further evaluation of the humanised slides intervention could involve investigations of how the balance of diversity displayed impacts students’ assumptions of diversity in their discipline.

### Practical considerations of using the humanised slides as an educational practice

Although our data indicate that the humanised slide design has a positive impact on student perceptions of diversity in science, we recognise that this intervention is insufficient to address the systematic biases within STEM. Our data suggests that presentation of humanised powerpoint slides in a single undergraduate lecture has some positive impact on student perceptions of diversity and, therefore, who belongs in the scientific community. It is important to note that we do not see the relatively modest number of students for whom the intervention was impactful as an indictment of its efficacy. Even if only one student’s perception of their own place within the field or their assumptions about who belongs in STEM changes positively as a result of this intervention, it will have had a meaningful impact. However, this intervention should be seen as a very small part in a wider catalogue of interventions that can be used to raise visibility of diversity in STEM [30, 52, 56]. The use of humanised slides is designed as a simple pedagogy that could be implemented by anyone, although there is a time-cost associated with finding and incorporating the pictures particularly. We see it as an important precursor to the long-term, and much slower, efforts to raise STEM diversity in textbooks, faculty membership, and wider portrayals of scientists in education and the media. There is also a danger that isolated attempts to improve diversity are seen as irrelevant or tokenistic, as it is known that poorly designed mandatory equity, diversity, and inclusion interventions are ineffective or can even backfire and create resentment or workplace tension [70]. For this practice to have sustained positive change for the majority of students, it needs to be used repeatedly and consistently through a programme by multiple teaching staff. It must also be accompanied by other active efforts to improve diversity and representation including hiring practices, inclusive curriculum design, appropriate mentoring schemes, and decolonization of the curriculum. A genuinely inclusive STEM curriculum would make space to actively discuss equity and diversity from a variety of perspectives going beyond gender and ethnicity to include non-binary and trans, disability, socioeconomic class, and international viewpoints [56]. Relying entirely on the humanised slide design is insufficient, as students may still make assumptions from photographs and names, and photographs cannot capture hidden aspects of diversity such as sexual orientation or non-visible disabilities. This approach is not the only way to increase representation and will be complemented by a range of other strategies. We would also encourage the inclusion of alumni or expert lecturers from diverse backgrounds in teaching, as well as scientist spotlight assignments [30], and other curriculum efforts to improve diversity [56].

It should also be noted that the humanised slide design practice is also only effective if instructors actively reflect on the studies they are including and make positive efforts to improve representation within their teaching materials. If the only studies presented in this format are written by older white men, then this practice could reinforce a perspective that practising scientists do not come from a diverse range of backgrounds. As academics, we have found that preparing slides in this format has prompted us to reconsider the studies we include in lectures, and actively seek out papers with a more diverse authorship. As such, we feel that the practice is also of benefit to instructors who are trying to make a positive difference but feel unsure of where to start. It should be noted that while designing slides in this format is a relatively modest intervention, it does increase the time spent on preparing teaching materials. We have found that selecting literature with appropriate authorship and obtaining images of researchers through searches on GoogleScholar or institutional websites does take time, but consider the activity to be of sufficient benefit. We recommend this as a straightforward way that academics in any setting, including those from majority demographic groups, can improve representation in a discipline relevant format.

## Conclusions and recommendations for practice

In this study, we conclude that students make assumptions about diversity of scientists from Harvard style references that reinforce biases developed by a lack of representation elsewhere in their education and development. We also provide initial evidence that presentation of photographs and full names of scientists alongside formal citations can have a positive impact on some student’s sense of belonging and encourage students to reflect on their biases about who belongs in STEM. We recommend adopting a humanised slide design in research-led teaching materials, so that students can have a better appreciation of the actual diversity of practising scientists that is masked by the formal citation. In our experience, adopting this slide design also actively encourages instructors to seek out papers with a diverse authorship. This is a straightforward intervention that all academics could make to give better representation to a diversity of scientists without having to find additional space within the curriculum. While this intervention is not sufficient to address all issues around diversity in STEM, it is an easy strategy that improves representation in an authentic way through research-led teaching, and may ultimately have a positive impact on the diversity of students choosing a scientific career.

## Supporting information

S1 File (DOCX)

S1 Data (XLSX)
