# Supplementary material for: I am a scientist: Overcoming biased assumptions around diversity in science through explicit representation of scientists in lectures
Source: PLoS One. 2023 Jul 7;18(7):e0271010. doi: 10.1371/journal.pone.0271010 (PMC10328340; doi:10.1371/journal.pone.0271010)
Supplement: S1 File — (DOCX) [file pone.0271010.s001.docx]

### Supplement A - Survey and coding methods

##### Supplemental Table A - Survey questions and answer format

| **Section 1: Completed prior to the taught session** | | | | |
| --- | --- | --- | --- | --- |
| Q1. Please circle which one of the following best describes you: | | | | |
| I study biology | I enjoy biology | I do biology | I am a biologist | |
| Q2. To what extent do you agree with the following statement: “Someone like me can be a biologist” | | | | |
| Strongly Agree | Agree | Neutral | Disagree | Strongly Disagree |
| Q3. To what extent do you agree with the following statement: “I want to be a biologist” | | | | |
| Strongly Agree | Agree | Neutral | Disagree | Strongly Disagree |
| Q4. Look at the reference below, what is your first instinct on what the author looks like, their first name, and where they come from? Lee, M. (2019) ‘Globally interesting Biology’. Biology Journal: 45 p. 12-25. [Open Response] | | | | |
| **Section 2: Completed after the taught session** | | | | |
| Q5. Did you notice the pictures and full-names of the scientists in the lecture [yes or no]? | | | | |
| Yes | | No | | |
| Q6. If yes, has it changed your perspective on Questions 2, 3 or 4 (please elaborate)? [Open Response] | | | | |
| Q7. Is this the first time you have seen pictures and full-names of the scientists being talked about in lectures [yes or no]? | | | | |
| Yes | | No | | |
| Q8. Do you feel this is good practice for lectures (please elaborate)? [Open Response] | | | | |
| Optional request for gender and ethnicity data | | | | |

####

#### Survey development

The survey used a pre- / post- model surrounding each taught session.

In the ‘Pre’ part of the survey, participants were asked a series of questions designed to investigate their ‘science identity’ and our implicit bias test (designed around a hypothetical Harvard reference). Our implicit bias test asked participants to share their first impressions on the identity of the author of Lee, M. (2019) ‘Globally interesting Biology’. *Biology Journal*: 45 p. 12-25. ‘M. Lee’ was chosen specifically to allow for multiple and diverse interpretations about the author the author as the surname Lee is common in communities descended from Anglophone, Korean, and Chinese ethnicities. We acknowledge that this choice of name ‘points’ towards these locations/ethnicities, and is less likely to be interpreted as e.g. an Arabic or African name. However, a truly globally neutral name does not exist, so we use “Lee” as a name that has feasible placement within multiple locations across the globe. To verify the neutrality of the initial ‘M’ we used the names dataset of Tzioumis (2018) and coded all 358 names beginning with M as either Male, Female or Gender Neutral. After weighting for frequency, 56% of names were Female, 44% were Male and less than 1% were Gender Neutral, indicating an approximately even distribution of gendered ‘M’ names. Within the Anglicised ‘M’ names there were both male and female Western names (e.g. Mark, Mary), Eastern European (Maciej, Mihaela), Indian (Madhu, Manjula), Islamic/Arabic (Muhammed, Maha) and East Asian names (Myung, Mei). While the Tzioumis (2018) dataset is drawn from American sources and, therefore, has a Western bias towards it, the diversity and gender balance of ‘M’ names indicates that the initial ‘M’ allows for multiple interpretations and does not perpetuate significant gender or nationality/ethnicity bias. It is worth noting that the questionnaire responses included ethnicities found in both Western and Eastern hemisphere, and both male and female first names were suggested. This supports the idea that the name ‘M Lee’ allowed for multiple gender and nationality/ethnicity interpretations.

Tzioumis, K. (2018) ‘Demographic aspects of first names’, *Scientific data*, 5, p. 180025. doi:10.1038/sdata.2018.25.

The ‘Post’ aspect of the survey was only available after the taught session and first asked whether the participants noticed “the pictures and full-names of the scientists in the lecture materials?”. If they answered yes, they were then asked whether the practice had changed their perspective on the questions asked before the taught session began. While this did include the questions designed to investigate participants’ sense of belonging (Q.1-3), participants’ responses were overwhelmingly focused on Q4. The survey questions related to student perception of the practice were similarly blunt and unequivocable. We asked “Do you feel this is good practice for lectures (please elaborate)?”; after reminding students what the practice was exactly in Q7. In this instance, we were specifically interested in student perceptions of the intervention because perceptions are one of the most significant barriers to engagement with diversity-positive educational interventions [[65]](https://paperpile.com/c/raUVNY/vitJQ). Note that we did not ask the question specifically in the context of diversity-positive education, and participants were free to outline positive and negative responses entirely unrelated to the visibility of diversity in STEM.

#### Thematic analysis of responses

This report focuses on participant responses to the three questions on the survey that had open text responses, which relate to research questions 1-3 respectively. The thematic coding process was completed separately for each question, and each will be outlined in detail below. Importantly, to allow for quantitative comparisons a system whereby each response could only be coded into a single theme was developed. This design was adopted to align to assumptions of statistical tests for count-based categorical variables (e.g. Chi-square test).

The first question to undergo thematic analysis was the student perceptions of the author of a hypothetical Harvard reference. The process started with an initial review of the participant responses to collate the most common kinds of assumption about the author. The four most consistently assumed characteristics were gender/sex (hereafter referred to as gender but recognising that these terms are not wholly interchangeable[[1]](https://paperpile.com/c/raUVNY/ULtVZ)), geographic location, ethnic group, and age; which became the four primary coding themes. Then every response was reviewed for comments relating to these four characteristics and was coded into one of: Bias, Reverse-Bias, Not Considered, and Diversity Aware. A ‘Not Considered’ code meant that the participant had made assumptions about other characteristics but not the specific one being coded (e.g. made assumptions about age but not ethnic group, so was scored as ‘not considered’ for ethnic group). ‘Diversity Aware’ answers either stated explicitly that one could not make assumptions about the author, were sincerely unsure of what to say, or made a humorous comment that made absolutely no assumptions about the author. Note it is possible that a response was coded as ‘Diversity Aware’ for one characteristic but biased for another characteristic if they stated that it is not possible to make assumptions but then in fact made implicit assumptions (e.g. “There isn't a first instinct, name can't be used to distinguish what someone looks like or where they come from. He's just as likely to be a white american as he is to be a black african”).

With respect to bias and reverse bias codes:

- Gender identity was coded either from a direct statement of sex(e.g. male = Bias), gender (e.g. man = bias), the use of gender-specific pronouns (e.g. she/hers = Reverse Bias), or according to the proposal of an explicitly and obviously gender-specific name (e.g. Mike, Martin, etc.).
- For ethnic group, responses linked to ‘white’ or caucasian were coded to Bias, while any other ethnic group(most commonly Chinese) was coded to Reverse-Bias.
- For location, explicit statements of West / Western / Developed were code to Bias, as were any European, UK, or North American locations. The few Australia responses were also included in the Bias theme. Any other location was considered to be Reverse Bias (most commonly China)
- Geographic location and ethnic groupwere separated by the statement of a place compared to the use of an ethnic group(e.g. China is a ‘Reverse Bias’ location vs. Chinese is a ‘Reverse Bias’ ethnic group). Ethnic groupwas not assumed from location (e.g. it was not assumed that the participant thought the author was Chinese if they stated the location as China). Where an ethnic group implicitly tied to a location was used without further clarification of location, then location was inferred (e.g. Chinese would be coded as reverse-bias for ethnic group and location).
- Biased age was coded from explicit statements that the author was middle-aged, old, bald, or grey-haired, while reverse-bias was coded in the one instance a participant assumed the author was ‘young’.

The second question responses for thematic analysis was Question 6 - “has it [humanised slides] changed your perspective on Questions 2, 3 or 4 (please elaborate)?”. The format of the question meant that the majority of participants started their response with a clear Yes or No answer, which was used as the initial basis for coding. ‘Yes’ responses were coded as ‘Explicit Change’; there were no ‘Yes’ responses that suggested that perceptions of diversity had become ‘worse’ as a result of the intervention. ‘No’ responses were placed into the ‘Explicit no change’ theme, unless the participant had elaborated to explain that the ‘No’ response was because they had assumed that the field was diverse already; coded as ‘Diversity Assumed’. However, not all responses had a clear ‘Yes’ or ‘No’ preface. So responses that included any explicit expression of a positive impact of the intervention on their perception of diversity or their sense of belonging within the field were coded as ‘Explicit Change’ (e.g. “*It's changed my perspective on 4 because I assumed it would be a white man but they were super diverse*”). Conversely, any response that highlighted a negative perception of the intervention, such as it being irrelevant or unnecessary, was coded as ‘Explicit no change’. Responses that explicitly stated no change without using the word ‘no’ were also coded to ‘Explicit no change’. There were two responses where the respondent repeated their answers to the previous questions instead of providing an answer to the specific question. One response was an exact repeat of the answers they provided for the previous questions, which was coded as explicit no change. The other indicated they had changed their perception on Question 3 from Agree to Strongly Agree, and this was coded as Explicit change. The final code allocated was No change described, which was linked to responses that highlighted both positive and negative elements, anyone who was unsure of the impact, or a response that was unrelated to the question. Once the highest level themes were coded each was reviewed separately for similarities in the rationale for participant answers. These sub-themes tied together the different types of reason the participants had given for answering the way that they had. Responses could be coded into multiple sub-themes, particularly where the open text response was long and comprehensive. The sub-themes that arose are considered in detail in the results section.

##### Supplementary Table B - Examples of responses to a question asking whether the intervention had changed student perspectives to the ‘pre-’ taught session questions, focusing particularly on how responses were coded into each theme.

| Code | Example quote | N number of respondents |
| --- | --- | --- |
| ‘Explicit positive change’ [starting with a ‘yes’] | “*Yes, I especially notices [sic] how young the majority of them were, which surprised me in a good way*”  *“Yes - it highlights the presence of 'non-cis white males' also publishing papers”* | 4 |
| ‘Explicit positive change’ [without a yes] | “*Slightly. Females are a part of science/biology more than we realise*”  “*It's changed my perspective on 4 because I assumed it would be a white man but they were super diverse*” | 14 |
| ‘Explicit no change’ [starting with a ‘no’] | “*No - I don't understand the importance or relevance of having academic headshots on lecture slides - we have the name and study if we want to look into them further but we have no need to know what they look like*”  “No, majority were still male (although more diverse) -> mainly asain” | 25 |
| ‘Explicit no change’ [without a ‘no’] | “*It has not changed*”  *“The Author is likely Male, Bald, uses glasses, probably in his late 50's whose first name is Micheal. Born from a Chinese family that migrated to United Kingdom”* - where this was identical to their answer to question 4  “*Q2 + Q3 +Q4 unchanged*” | 8 |
| Change not described | “*Not going to lie I was too busy laughing at the first guy called Pu Tang*”  “*I didn't see Mr Martin Lee from Bristol*” | 10 |
| Diversity Assumed | “*No, I already had the mindset that it would be a wide range of people carrying out the research*”  “i don't think they would of changed my answer because i dont really have a set idea anyway” | 16 |

The final thematic analysis process was for responses to Question 8 - “Do you feel this is good practice for lectures (please elaborate)?”. Similarly to the previous question, most answers began with a ‘Yes’ or ‘No’ response, which provided the first level of coding. ‘Yes’ responses were automatically coded into the theme ‘Explicit good practice’, while ‘No’ responses into ‘Explicit not good practice’. In a few cases, this was the whole of the answer. Not all questions started with a ‘Yes’/’No’ statement, in these instances the response was reviewed for value-statements that were inherently positive or negative. Responses that were deemed to be unambiguously positive were coded to Explicitly good practice, while those that were negative were coded to ‘Explicit not good practice’. These were generally clear but conversations with the second coder were used to ensure consistency of response. The final code allocated was Ambivalent, which was linked to responses that highlighted both positive and negative elements, anyone who was unsure of the impact, or a response that was unrelated to the question. Once the highest level themes were coded each was reviewed separately for similarities in the rationale for participant answers. These sub-themes tied together the different types of reason the participants had given for answering the way that they had. Responses could be coded into multiple sub-themes, particularly where the open text response was long and comprehensive. The sub-themes that arose are considered in detail in the results section.

##### Supplementary Table C - Examples of responses to a question asking whether the participant felt that intervention was good practice, focusing particularly on how responses were coded into each theme.

| Code | Example quote | N (number of responses) |
| --- | --- | --- |
| ‘Explicit good practice’ [starting with a ‘yes’] | “*Yes, it's nice to see people like me!*”  *“Yes as it shows the diversity of real scientists and it helps to prevent prejudice against certain groups of scientists when reading scientific papers later in life”* | 59 |
| ‘Explicit good practice’ [without a yes] | “*Good idea helps to picture the person and their experiences*”  *“Maybe it could encourage people as they can see themselves represented, also pictures make things more interesting”* | 9 |
| ‘Explicit not good practice’ [starting with a ‘no’] | “*No, prefer to have the links to the paper so I can read them if I want*”  *“no, i feel like knowing someone’s name/gender/ethnicity creates bias towards the paper”* | 7 |
| ‘Explicit not good practice’ [without a ‘no’] | “*I would rather focus on the information rather than the author*”  “In my opinion what the scientist looks like is irrelevant to the legitimacy of their work, however showing who the scientist is in cases where a certain person is known to present incorrect information is important” | 7 |
| Ambivalence  [mixed or neutral] | “*Interesting but unsure of relevance*”  *“Neutral opinions as I don't focus much on it”* | 4 |
| Ambivalence  [unsure] | “Maybe”  “I don’t mind, sure” | 5 |

References

1 [Newman T. Sex and gender: Meanings, definition, identity, and expression. 11 May 2021 [cited 9 Sep 2022]. Available:](http://paperpile.com/b/raUVNY/ULtVZ) <https://www.medicalnewstoday.com/articles/232363#identity-and-expression>
